# Neuroinflammation and protein pathology in Parkinson’s disease dementia

**DOI:** 10.1186/s40478-020-01083-5

**Published:** 2020-12-03

**Authors:** Antonina Kouli, Marta Camacho, Kieren Allinson, Caroline H. Williams-Gray

**Affiliations:** 1 John van Geest Centre for Brain Repair, Department of Clinical Neurosciences, University of Cambridge, Cambridge, UK; 2 Department of Pathology, Cambridge University Hospitals NHS Foundation Trust, Cambridge, UK

**Keywords:** Parkinson’s disease dementia, Neuropathology, Neuroinflammation, Microglia, Infiltrating lymphocytes, Pro-inflammatory cytokines, Toll-like receptors

## Abstract

Parkinson’s disease dementia is neuropathologically characterized by aggregates of α-synuclein (Lewy bodies) in limbic and neocortical areas of the brain with additional involvement of Alzheimer’s disease-type pathology. Whilst immune activation is well-described in Parkinson’s disease (PD), how it links to protein aggregation and its role in PD dementia has not been explored. We hypothesized that neuroinflammatory processes are a critical contributor to the pathology of PDD. To address this hypothesis, we examined 7 brain regions at postmortem from 17 PD patients with no dementia (PDND), 11 patients with PD dementia (PDD), and 14 age and sex-matched neurologically healthy controls. Digital quantification after immunohistochemical staining showed a significant increase in the severity of α-synuclein pathology in the hippocampus, entorhinal and occipitotemporal cortex of PDD compared to PDND cases. In contrast, there was no difference in either tau or amyloid-β pathology between the groups in any of the examined regions. Importantly, we found an increase in activated microglia in the amygdala of demented PD brains compared to controls which correlated significantly with the extent of α-synuclein pathology in this region. Significant infiltration of CD4^+^ T lymphocytes into the brain parenchyma was commonly observed in PDND and PDD cases compared to controls, in both the substantia nigra and the amygdala. Amongst PDND/PDD cases, CD4^+^ T cell counts in the amygdala correlated with activated microglia, α-synuclein and tau pathology. Upregulation of the pro-inflammatory cytokine interleukin 1β was also evident in the substantia nigra as well as the frontal cortex in PDND/PDD versus controls with a concomitant upregulation in Toll-like receptor 4 (TLR4) in these regions, as well as the amygdala. The evidence presented in this study show an increased immune response in limbic and cortical brain regions, including increased microglial activation, infiltration of T lymphocytes, upregulation of pro-inflammatory cytokines and TLR gene expression, which has not been previously reported in the postmortem PDD brain.

**Electronic supplementary material:**

The online version of this article (10.1186/s40478-020-01083-5) contains supplementary material, which is available to authorized users.

## Introduction

The development of dementia is a key milestone in the progression of Parkinson’s disease (PD). Almost half of patients develop PD dementia (PDD) within 10 years from diagnosis [76], reaching over 80% at 20 years [29]. Widespread cortical and limbic Lewy body deposition has been reported by several clinicopathological studies to be the best pathological correlate of cognitive decline in PD [1, 30, 34, 38, 47, 51]. However, the association between cortical Lewy body pathology and PD dementia is far from clear-cut, given that approximately one-third of PD cases classified as Braak PD Stage 3 (indicative of no neocortical Lewy bodies) were found to be demented during life [5]. Conversely, a number of PD cases exhibiting neocortical and/or limbic Lewy body pathology had no history of cognitive impairment [8, 60]. Other studies have reported a significant role for co-existing Alzheimer’s type pathology [10, 27, 37, 39, 41, 64] and a combination of both cortical Lewy body and Alzheimer’s-type pathologies has been suggested as a more robust correlate of PD dementia [9, 31]. Despite extensive research on the neuropathological substrate of PDD, a consensus has yet to be reached. These conflicting results may be in part due to differences in case selection, the methodologies used, as well as the inherent heterogeneity of the disease, however, it also suggests that mechanisms other than protein aggregation may be critically contributing to cognitive decline in PD.

Neuroinflammation in the PD brain has been described in a small number of postmortem studies, as well as in vivo using [^11^C]PK11195 PET imaging [25], but clinicopathological studies assessing neuroinflammation in PD dementia cases are lacking. McGeer and colleagues were the first to report an increase in the number of HLA-DR^+^ microglia in the substantia nigra of PD compared to healthy brains [52], while a subsequent study found increased numbers of microglia in the hippocampus, transentorhinal, cingulate and temporal cortices of PD cases compared to controls [36]. Infiltration of both helper (CD4^+^) and cytotoxic (CD8^+^) T lymphocytes into the parenchyma of the substantia nigra has been observed in the vicinity of neuromelanin-positive dopaminergic neurons in the PD brain [6]. Upregulation of pro-inflammatory cytokines has also been reported in PD, including increased expression of tumour necrosis factor α (TNFα), interleukin 1β (IL-1β), and interferon γ (IFNγ) in the substantia nigra, and upregulation of interleukin 6 (IL6) and interleukin 2 (IL2) in the striatum [33, 55–57]. Pro-inflammatory cytokine expression has not been explored in more widespread brain regions.

A prominent pathway regulating inflammatory responses is mediated by Toll-like receptors (TLRs) [42]. Accumulating evidence suggests that α-synuclein may be triggering microglial activation via TLR2 and TLR4, leading to downstream secretion of pro-inflammatory mediators [13, 22, 44, 46]. Both these receptors have been found to be upregulated at the protein level in the caudate/putamen of postmortem PD cases compared to controls [16], with TLR4 also being elevated in the substantia nigra [67] and TLR2 in the anterior cingulate cortex [19].

Hence, although there is accumulating evidence suggesting that neuroinflammation is a feature of the pathology of PD, investigation of neuroinflammatory processes in extra-nigral brain regions has been limited to date, and no studies have explored associations with cognitive status during life. We therefore sought to characterize inflammatory changes across multiple brain regions in demented compared to non-demented PD cases and age-matched controls, in addition to better characterizing the anatomical pattern of misfolded protein pathology in these cases. We explored the relationship between markers of neuroinflammation and aberrant forms of α-synuclein, tau and amyloid-β, as well as the association of neuropathological findings with cognitive decline during life.

## Materials and methods

### Human samples

This study received ethical approval from the London—Bloomsbury Research Ethics Committee (16/LO/0508). Postmortem brain tissue from 28 idiopathic PD cases and 14 age and sex-matched controls with no known history of neurological or neuropsychiatric symptoms was acquired from the Queen’s Square Brain Bank and the Cambridge Brain Bank. Presence/absence of tau or amyloid-β pathology was not used as a selection criterion for controls, as our aim was the comparison of PD cases with typical neurologically healthy aged individuals in whom a degree of incidental protein pathology is expected. Brains were bisected in the sagittal plane with one half flash-frozen and stored at − 80 °C and the other half fixed in 10% neutral buffered formalin for 2–3 weeks. From the formalin-fixed tissue, blocks were sampled and embedded in paraffin.

All PD cases had been assessed during life at the Parkinson’s Disease Research Clinic, University of Cambridge, UK, with prospective collection of longitudinal clinical and neuropsychological data. All PD cases met the UK Parkinson’s Disease Society Brain Bank Diagnostic Criteria. Cause of death was determined based on the death certificate. Standardized assessments included the Unified Parkinson’s Disease Rating Scale (UPDRS), Hoehn and Yahr stage, the Mini-Mental State Examination (MMSE), and verbal fluency testing. PD dementia was diagnosed using MDS PD Dementia level 1 criteria [17, 21], operationalized using MMSE < 26, impaired cognitive performance in more than 1 domain and impairment in functional ability on activities of daily living as assessed by the clinician. In cases who were lost to follow-up from the Parkinson’s Disease Research Clinic, dementia status was determined retrospectively through review of the medical notes.

### Immunoperoxidase staining of human postmortem brain tissue

Immunohistochemistry was performed on 10 μm-thick paraffin-embedded sections from 7 brain regions: substantia nigra, amygdala, hippocampus, entorhinal, occipitotemporal, prefrontal, and posterior parietal cortex. One section per brain region was stained for each marker of interest in control and PD cases. Sections were deparaffinized and sequentially rehydrated, in xylene, 100% EtOH, 90% EtOH, 70% EtOH and dH_2_O. Antigen retrieval was performed in 98% formic acid (pH = 1.6–2.0) for 5 min (α-synuclein and tau), or in boiling 10 mM sodium citrate buffer (0.05% Tween20, pH = 6) for 30 min (all other antibodies). Blocking of endogenous peroxidase activity was performed in 3% H_2_O_2_ in PBS, for 15 min at room temperature. Sections were then incubated with blocking solution (2% milk for α-synuclein and tau, or 20% normal rabbit serum for all other antibodies) for 20 min at room temperature. Sections were subsequently incubated with the appropriate primary antibody for 1 h at room temperature (α-synuclein [Enzo Life Sciences sa3400, 1:250]; tau [in house P11/57 clone, 1:5]; amyloid-β [DAKO MO872, 1:100]; GFAP [DAKO Z0334, 1:500]; HLA-DR [DAKO MO775, 1:500]; Iba1 [Wako Chemicals 019-19741, 1:4000]; CD3 [Leica Biosystems NCL-L-CD3-565, 1:300]; CD4 [Abcam ab133616, 1:100]; CD8 [Abcam ab17147, 1:100]). Following 3 × 5 min washes with PBS, sections were incubated with biotinylated secondary antibody for 30 min at room temperature. Following 3 × 5 min washes with PBS, the sections were incubated with ABC Elite Vectastain Kit (Vector Laboratories) for 30 min at room temperature. Colour was developed by 4 min incubation in DAB Peroxidase Substrate solution (Vector Laboratories). Upon rinsing with dH_2_O, sections were counterstained with Harris’ Haematoxylin for 30 s, sequentially dehydrated in ascending EtOH concentrations and coverslipped using DPX mounting medium. Slide scanning was done at the Histopathology/HIS facility at the Cancer Research UK Cambridge Institute. Scanning was performed on the Aperio Scanscope AT2 (Leica Biosystems) at ×20 magnification with a resolution of 0.503 μm per pixel. Images were viewed with the Aperio Imagescope viewing platform (Leica Biosystems).

### Neuropathological assessment

The diagnosis of idiopathic PD was confirmed by the presence of Lewy bodies in the substantia nigra. Neuropathological assessment of α-synuclein, tau, and amyloid-β was performed using Aperio ImageScope software. Lewy body, neurofibrillary tau tangle and amyloid-β pathology were assessed by a recently described quantitative method based on a digital analysis package in ImageScope [18]. Specifically, the Positive Pixel Count v9 algorithm was used with parameters optimized for the quantification of brown (DAB) immunohistochemical staining. To account for differences in ROI size across postmortem cases, the specific staining is reported as the total positive pixels per mm^2^ stained area. Microglia were counted by a quantitative semi-automated method using ImageJ Software (Rasband, W.S., ImageJ, U. S. National Institutes of Health, Bethesda, Maryland, USA). Specifically, 3 × 1 mm^2^ square ROIs were randomly selected from each brain region. Image processing included the following steps: colour deconvolution, 8-bit conversion, background subtraction and noise reduction. The appropriate brightness threshold was manually determined to create an overlay mask. The particle analysis plugin was used to count the number of activated microglia per mm^2^. Size settings were optimized to primarily include enlarged amoeboid (activated microglia) and not small, ramified cells. This was done by measuring the cell soma diameter of amoeboid microglia in several sections in different brain regions and then using this experimenter-determined diameter in the particle analysis plugin to quantify cells of similar or larger cell soma size only. This algorithm was applied to all the images across all brain regions using the Multiple Image Processor plugin and the mean of the 3× ROIs was used for statistical analysis. The same methodology was used for the quantification of HLA-DR^+^ and Iba1^+^ activated microglia. Astroglial quantification was done by a semi-automated method using ImageJ Software. The total GFAP stained area was measured in 3 × 1 mm^2^ square ROIs per brain region and the mean was used for statistical analysis. Finally, the number of parenchymal but not perivascular CD4^+^ and CD8^+^ T lymphocytes was manually counted in the entire section of the substantia nigra and the amygdala. Infiltrating lymphocytes were expressed as the number of cells per mm^2^.

### RNA extraction from frozen postmortem brain

Frozen tissue samples from the substantia nigra, amygdala, hippocampus, and frontal cortex were used for mRNA extraction. 20–40 mg of tissue were homogenized with Qiazol Lysis Reagent. RNA was then purified using the RNeasy Plus Universal Mini Kit according to the manufacturer’s instructions. RNA concentration was measured using a NanoDrop spectrophotometer. RNA integrity was determined by Agilent 2100 Bioanalyzer using Agilent RNA 6000 Nano Chips according to the manufacturer’s instructions. RNA samples with RIN numbers ≤ 5 were not used for further analysis. 300 ng of total RNA was converted to cDNA using the SuperScript™ III First-Strand Synthesis SuperMix for qRT-PCR as per the manufacturer’s instructions.

### Real-time quantitative PCR

For each cDNA sample, the 20 µl reaction mixture consisted of 10 µl of TaqMan Gene Expression Mastermix (Thermo Fisher Scientific #4369510), 1 µl of the appropriate TaqMan primer/probe, 4 µl DNase free H_2_O and 5 µl cDNA. A non-template sample (containing TaqMan primer/probe, TaqMan master mix and DNase free H_2_O without cDNA) was used as a negative control. The following TaqMan (Thermo Fisher Scientific) primer/probes were used: TLR2 (Hs02621280_s1), TLR4 (Hs00152939_m1), TNFα (Hs00174128_m1), IL-1β (Hs01555410_m1), IL6 (Hs00174131_m1), IL8 (CXCL8) (Hs00174103_m1), and the housekeeping reference genes CYC1 (Hs00357717_m1), UBE2D2 (Hs00366152_m1) and GAPDH (Hs04420697_g1). Real-time amplifications were run in triplicates in a QuantStudio™ 12 K Flex Real-Time PCR System (Applied Biosystems). The reaction mixtures were incubated at 50 °C for 2 min and 95 °C for 10 min followed by 40 cycles at 95 °C for 10 s, and 60 °C for 1 min. The expression of the target genes (C_T_) was normalized by subtracting the mean C_T_ of three housekeeping reference genes (CYC1, UBE2D2 and GAPDH) giving the ΔC_T_ for each sample. Statistical analysis was done on the ΔC_T_ values as recommended by Yuan et al. [78] using two-tailed unpaired t-tests. For the graphical representation of the fold-change, the Livak method was used [49]. Briefly, the mean ∆C_T_ of the control group was subtracted from the ∆C_T_ of each sample to get the ∆∆C_T_. Finally, the formula 2^−∆∆CT^ was used to extrapolate the fold-change. Fold-change is 1 for the control group (no change). For the PD group, fold-change > 1 indicates increased gene expression, whereas < 1 denotes decreased expression compared to the control group.

### Statistical Analysis

The Shapiro–Wilk normality test was used to assess the distribution of variables. Accordingly, comparisons between control, PDND and PDD groups, were performed either with a one-way ANOVA or a Kruskal–Wallis test (for parametric and nonparametric variables, respectively), whilst correcting for multiple comparisons with the appropriate post hoc test. Comparisons between two groups were made with an unpaired two-tailed *t* test or by Mann-Whitey U test (for parametric and nonparametric variables, respectively). Categorical variables were compared using a χ^2^ test.

Spearman’s rank-order correlation was used to assess correlations between neuropathological variables within specific regions. The association of pathological variables with cognitive decline during life (change in MMSE per year) was first assessed by Spearman’s rank-order correlation. Pathological variables found to be associated with cognitive decline (*p* < 0.05) were then entered into a univariate linear regression analysis, with rate of cognitive decline as the dependent variable, whilst correcting for age at death and disease duration. All correlative analyses (between pathological, immune, and clinical markers) were exploratory and so formal correction for multiple testing was not applied.

IBM SPSS Statistics and GraphPad Prism were used for statistical analysis. Graphs were generated using GraphPad Prism. A *p* value < 0.05 was defined as statistically significant. The data is presented as mean (± SD) unless otherwise specified.

## Results

### Demographics

Demographic and clinical characteristics of PD patients (n = 28) and controls (n = 14) are summarized in Table 1. 11 PD patients developed dementia during life (PDD), whilst 17 had remained cognitively intact (PDND). The mean duration of dementia from onset to death was 4.7 (± 2.9) years. Controls, PDND and PDD cases were matched for age, sex, and postmortem interval. For 5 of the PD cases, clinical data was only available for a single visit to the clinic. Those cases were excluded from the “interval from last assessment to death” and “change per year” analysis.

**Table 1:** Control and PD case demographics

	Control	PDND	PDD	*p value*
N	14	17	11	–
Postmortem interval (h)	64.1 (± 38.3)	58.8 (± 28.1)	61.5 (± 32.7)	0.903
Sex: % Male	64.3%	51.9%	90.9%	0.112
Age at death	79.8 (± 5.9)	79.0 (± 8.1)	81.8 (± 6.4)	0.573
Age at diagnosis	–	66.9 (± 11.1)	67.9 (± 9.7)	0.803
Disease duration at death (yr)	–	12.1 (± 4.8)	13.9 (± 6.4)	0.396
Interval from last assessment to death (yr)	–	4.2 (± 3.3)	3.9 (± 4.1)	0.749
Last Hoehn and Yahr score	–	2.6 (± 0.7)	2.9 (± 0.8)	0.693
Last UPDRS motor (on medication)	–	30.6 (± 15.0)	39.3 (± 9.9)	0.117
Last UPDRS total (on medication)	–	50.2 (± 21.3)	69.8 (± 16.9)	0.021*
Last MMSE	–	28.7 (± 1.2)	20.6 (± 5.1)	< 0.0001****
Change in MMSE per year	–	0.21 (± 0.64)	− 1.46 (± 1.34)	< 0.0001****

Continuous variables were compared using One-way ANOVA (between Control, PDND and PDD) or Mann–Whitney U test (between PDND and PDD). Categorical variables (sex) were compared using the χ^2^ test. *UPDRS* Unified Parkinson’s Disease Rating Scale, *MMSE* Mini-Mental State Examination, *PDND* Parkinson's disease no dementia, *PDD* Parkinson's disease dementia. The values represent the mean (± SD)

*p < 0.05, ****p < 0.0001

There was no difference in disease duration between the PDND and PDD groups. Total UPDRS at last assessment was significantly higher in PDD compared to PDND cases (*p* = 0.021) as anticipated, but PDND and PDD groups were matched in terms of motor severity (UPDRS-III) at last assessment. PDD cases had significantly lower MMSE scores at their last clinic visit prior to death, compared to PDND cases (*p* < 0.0001), as well as a significantly greater decline in MMSE scores per year (*p* < 0.0001).

**Fig. 1:**
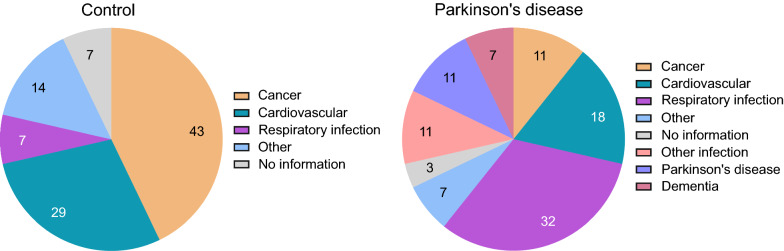
Primary cause of death in controls and Parkinson’s disease cases. Among those dying of cancer, primary sites were the oesophagus, pancreas, colon, liver, lung, skin, breast and endometrium. Respiratory infections included bronchopneumonia, aspiration sepsis and lower respiratory tract infection, while other infections were urinary sepsis and Staphylococcal septicaemia. Cardiovascular causes of death were acute myocardial infarction, ischaemic heart disease, cardiac arrest, pulmonary embolism and ruptured aortic aneurysm. “Other” in the control group included old age and multiple organ failure and in the PD group, chronic obstructive pulmonary disease, and acute respiratory failure

Cause of death was also interrogated in the controls and PD cases (Fig. 1). The most frequent primary cause of death in the control group was cancer (43%) followed by cardiovascular conditions (29%). In the PD group, death was predominantly due to respiratory infection (32%; mainly bronchopneumonia) and cardiovascular disease (18%), with cancer and Parkinson’s disease being the third most common causes (11% each). Dementia was recorded as the primary cause of death in 7% of PD cases.

### Increased cortical α-synuclein pathology, but not tau or amyloid-β in PDD compared to PDND brains

Examination of the substantia nigra confirmed marked loss of pigmented cells and the presence of Lewy bodies and Lewy neurites in the remaining pigmented cells in all PD cases, but not in any of the controls. Formal quantification of Lewy body counts in this region was of little value as they were confounded by cell death. No neurofibrillary tau tangles or amyloid-β plaques were identified in the substantia nigra in either controls or PD cases. The extent of α-synuclein, tau and amyloid-β pathology was quantitatively measured in a further six brain regions, namely the hippocampus, amygdala, entorhinal, occipitotemporal, prefrontal and posterior parietal cortices.

**Fig. 2:**
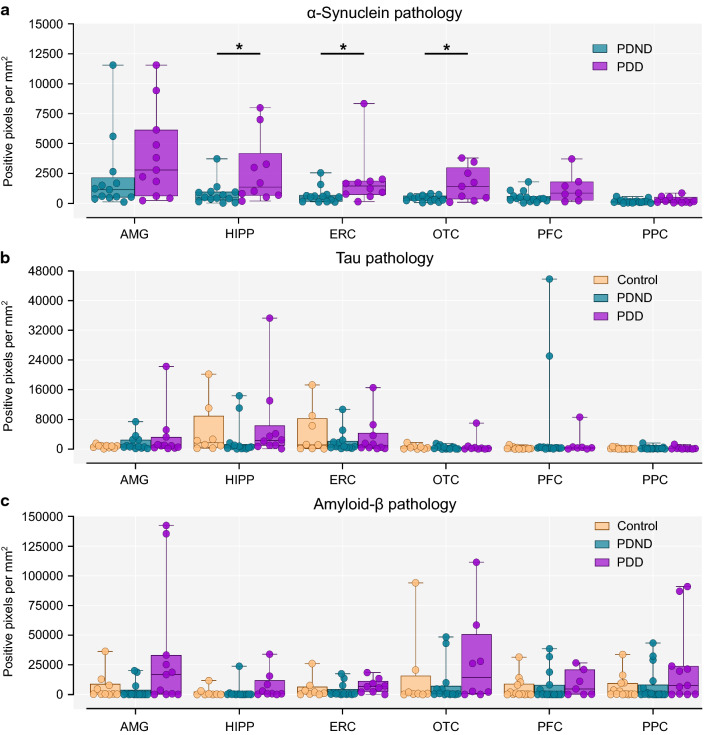
Quantification of α-synuclein, tau and amyloid-β pathology in multiple brain regions. **a** There was in increase in α-synuclein pathology in PDD compared to PDND cases in the hippocampus (Mann–Whitney U test, *p* = 0.015), entorhinal (Mann–Whitney U test, *p* = 0.015), and occipitotemporal cortex (Unpaired t test with Welch’s correction, *p* = 0.037). **b** There was no difference in tau pathology between groups in any region (Kruskal–Wallis test with Dunn’s correction for multiple comparisons, *p* > 0.05). **c** There were no between-group differences in amyloid-β pathology (Kruskal–Wallis test with Dunn’s correction for multiple comparisons, *p* > 0.05). AMG: Control n = 10, PDND n = 13, PDD n = 11, HIPP/ERC/OTC: Control n = 8, PDND n = 13, PDD n = 10, PFC: Control n = 13, PDND n = 15, PDD n = 7, PPC: Control n = 13, PDND n = 17, PDD n = 11. *PDND* Parkinson's disease no dementia, *PDD* Parkinson's disease dementia, *AMG* amygdala, *HIPP* Hippocampus, *ERC* entorhinal cortex, *OTC* occipitotemporal cortex, *PFC* prefrontal cortex, *PPC* posterior parietal cortex. *p < 0.05

Comparison of α-synuclein pathology was made only between PDND and PDD cases but not with controls due to the complete absence of Lewy bodies and neurites in the latter (Fig. 2a). α-Synuclein pathology was greater in PDD compared to PDND across multiple brain regions, with comparisons reaching significance (*p* < 0.05) in the hippocampus, entorhinal, and occipitotemporal cortex. There were no statistically significant differences in tau or amyloid-β pathology in any brain regions between the three groups (Fig. 2b, c).

### Increased astrogliosis but not microglial activation in the PD substantia nigra

**Fig. 3:**
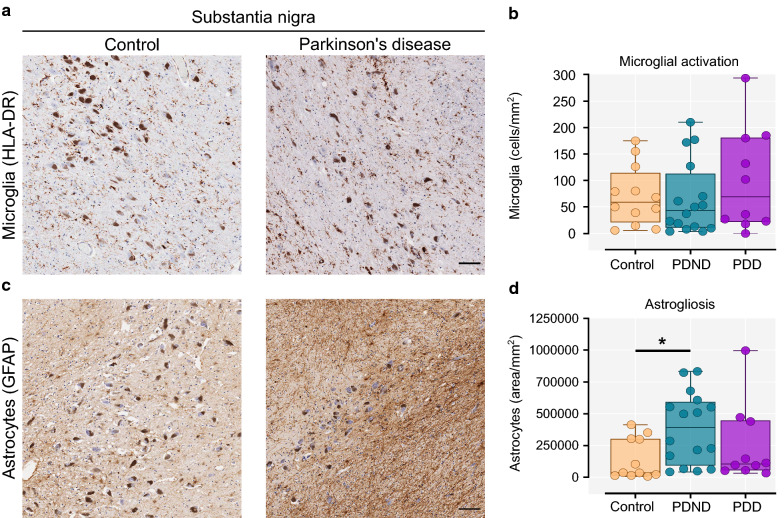
Microglial activation and astrogliosis in the substantia nigra. **a** Representative image of HLA-DR^+^ microglia in the substantia nigra of a control (left) and a Parkinson’s brain (right). The dark brown pigmented cells are neuromelanin-containing dopaminergic neurons. **b** Quantification of the total activated (enlarged amoeboid) microglia per mm^2^ (Kruskal–Wallis with Dunn’s multiple comparisons test, *p* = 0.646). **c** Representative image of astrocytic GFAP immunostaining in the substantia nigra of a control (left) and a Parkinson’s brain (right). **d** Quantification of the total GFAP-stained area per mm^2^ (Kruskal–Wallis with Dunn’s multiple comparisons test, *p* = 0.024; Control vs PDND *p* = 0.019). Control n = 12, PDND n = 16, PDD n = 10. *PDND* Parkinson’s disease no dementia, *PDD* Parkinson’s disease dementia. Scale bar: 100 μm. *p < 0.05

In the substantia nigra, quantification of the number of enlarged amoeboid HLA-DR^+^ microglial cells (activated) revealed no significant difference between controls, PDND and PDD brains (Fig. 3a, b). Given that this finding contradicts previous reports of an increase in activated microglia in the nigra in PD, we performed additional staining in this region using the microglial marker Iba1, which confirmed similar results (Supplementary Figure 1). Astrogliosis (GFAP^+^ stained area) was increased in PDND brains compared to controls (Kruskal–Wallis with Dunn’s multiple comparisons test, *p* = 0.024; Control vs PDND *p* = 0.019) (Fig. 3c, d). This suggests increased astrocytic scarring in this brain region which could be related to the widespread neuronal cell death.

### Microglial activation is increased in the amygdala of PDD cases

**Fig. 4:**
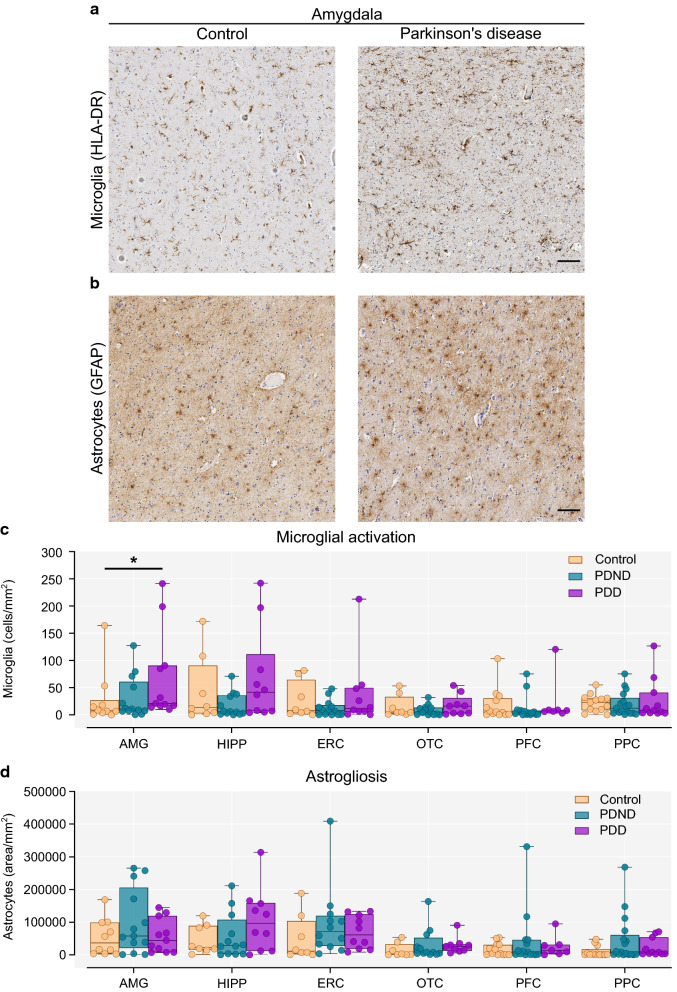
Microglial activation and astrogliosis in extra-nigral brain regions. **a** Representative image of HLA-DR^+^ microglia in the amygdala of a control (left) and a Parkinson’s brain (right). **b** Representative image of astrocytic GFAP immunostaining in the amygdala of a control (left) and a Parkinson’s brain (right). **c** Quantification of activated (enlarged amoeboid) microglia per mm^2^. There was a significant increase in the number of activated microglia in the amygdala of PDD cases compared to controls (Kruskal–Wallis with Dunn’s multiple comparisons test, *p* = 0.039; Control vs PDD *p* = 0.045). **d** Quantification of the total GFAP-stained area per mm^2^ (Kruskal–Wallis with Dunn’s multiple comparisons test, *p* > 0.05). AMG: Control n = 10, PDND n = 13, PDD n = 11; HIPP/ERC/OTC: Control n = 8, PDND n = 13, PDD n = 10; PFC: Control n = 13, PDND n = 15, PDD n = 7. PPC: Control n = 13, PDND n = 17, PDD n = 11. *PDND* Parkinson’s disease no dementia, *PDD* Parkinson’s disease dementia, *AMG* amygdala, *HIPP* hippocampus, *ERC* entorhinal cortex, *OTC* occipitotemporal cortex, *PFC* prefrontal cortex, *PPC* posterior parietal cortex. Scale bar: 100 μm. *p < 0.05

Quantification of microglial activation and astrogliosis was performed in six additional brain regions, namely, the amygdala, hippocampus, entorhinal, occipitotemporal, prefrontal and posterior parietal cortex. Activated microglia counts in the amygdala were significantly higher in PDD cases compared to controls (Kruskal–Wallis with Dunn’s multiple comparisons test, *p* = 0.039; Control vs PDD *p* = 0.046) (Fig. 4a, c). The number of activated microglia in the hippocampus was also higher in PDD compared to PDND cases, but this did not withstand multiple comparisons correction. The number of activated microglia did not differ between groups in the remaining brain regions (Fig. 4c). Astrogliosis did not differ between controls, PDND and PDD cases in any brain region (Fig. 4b, d).

### Increased T lymphocyte infiltration in PDND and PDD brains compared to controls

**Fig. 5:**
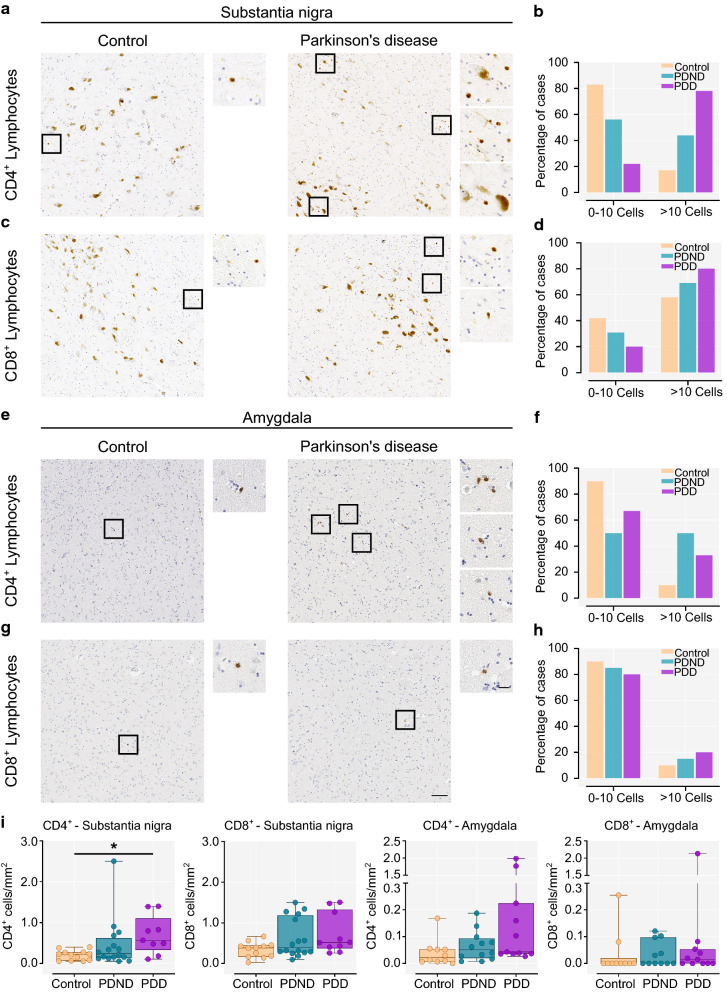
CD4^+^ and CD8^+^ T lymphocytes in the substantia nigra and the amygdala. **a**, **c** Representative image of parenchyma infiltrating CD4^+^ and CD8^+^ T lymphocytes in the substantia nigra of a control (left) and a Parkinson’s brain (right). The dark brown pigment is neuromelanin within dopaminergic neurons; the smaller CD4^+^ and CD8^+^ T cells are shown in the higher magnification inserts indicated by black squares. **b**, **d** Percentage of controls, PDND and PDD cases that show 0–10 or more than 10 infiltrating CD4^+^ (χ^2^ = 75.15, *p* < 0.0001) or CD8^+^ T cells (χ^2^ = 11.31, *p* = 0.0035) in the entire nigral section **e**, **g** Representative image of parenchyma infiltrating CD4^+^ and CD8^+^ T lymphocytes in the amygdala of a control (left) and a Parkinson’s brain (right). CD4^+^ and CD8^+^ T cells are shown in the higher magnification inserts indicated by black squares. **f**, **h** Percentage of controls, PDND and PDD cases that show 0–10 or more than 10 infiltrating CD4^+^ (χ^2^ = 37.68, *p* < 0.0001) or CD8^+^  T cells (χ^2^ = 3.92, *p* = 0.141) in the entire amygdala section. **i** Quantification of parenchymal CD4^+^ (*p* = 0.023, Control vs PDD *p* = 0.018) and CD8^+^ T lymphocytes (*p* = 0.102) per mm^2^ in the substantia nigra. Control n = 12, PDND n = 16, PDD n = 10. Quantification of parenchymal CD4^+^ (*p* = 0.081) and CD8^+^ T lymphocytes (*p* = 0.434) per mm^2^ in the amygdala. Control n = 10, PDND n = 12, PDD n = 11. Kruskal–Wallis with Dunn’s multiple comparisons test. *PDND* Parkinson’s disease no dementia, *PDD* Parkinson’s disease dementia. Scale bar: 100 μm. Scale bar (insert): 20 μm. *p < 0.05

Initial investigations in the substantia nigra in a subset of brains indicated a higher number of parenchyma infiltrating CD3^+^ T cells per mm^2^ in PD versus controls (*p* = 0.038). (Supplementary Figure 2). Parenchyma-infiltrating CD3^+^ T cells were also observed in the amygdala in both PD cases and controls, but these cells were extremely sparse or absent in the hippocampus and the cortical brain regions we investigated. Therefore, the substantia nigra and the amygdala were further examined quantitatively for the presence of CD4^+^ and CD8^+^ T lymphocyte infiltration. Representative images of CD4^+^ and CD8^+^ immunostaining in the substantia nigra and amygdala of a control and Parkinson’s case are shown in Fig. 5a and c. The percentage of postmortem cases with 0–10, or more than 10 infiltrating cells in the entire section is illustrated in Fig. 5b, d, f, and h.

In the substantia nigra only 17% of controls showed significant CD4^+^ lymphocyte infiltration (> 10 cells) compared to 44% of PDND and 78% of PDD cases (χ^2^ = 75.15, *p* < 0.0001) (Fig. 5b). Quantification of parenchyma-infiltrating lymphocytes per mm^2^ revealed a significant increase in the number of CD4^+^ T cells in the substantia nigra in PDD > PDND > controls (*p* = 0.016; Control vs PDD *p* = 0.014) (Fig. 5i). A larger proportion of PDD cases (80%) had more than 10 CD8^+^ T lymphocytes in the substantia nigra compared to PDND cases (69%) and controls (58%) (χ^2^ = 11.31, *p* = 0.0035) (Fig. 5d). However, there was no difference in the number of CD8^+^ T cells per mm^2^ across groups after correcting for multiple comparisons (*p* = 0.105) (Fig. 5i). In the amygdala, only 10% of controls showed marked CD4^+^ T lymphocyte infiltration (> 10 cells), as compared to 50% of PDND and 33% of PDD cases (χ^2^ = 37.68, *p* < 0.0001) (Fig. 5d). The number of CD4^+^ T cells per mm^2^, however, was not significantly different between the three groups after correcting for multiple comparisons (*p* = 0.081) (Fig. 5i). The number of infiltrating CD8^+^ T lymphocytes in the amygdala was comparable across groups (*p* = 0.434) (Fig. 5i).

### Pro-inflammatory cytokine IL-1β expression is upregulated in the substantia nigra and frontal cortex of PD brains

**Fig. 6:**
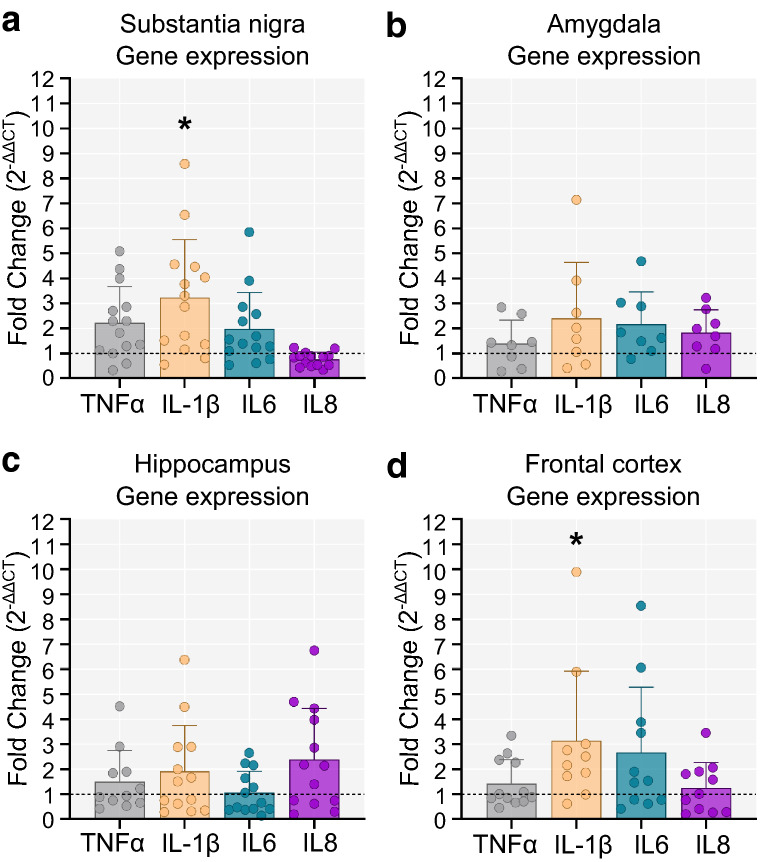
Expression of inflammatory cytokines in PD and control brains. Fold change in cytokine gene expression in the PD group relative to the control group. The dotted line marks the control group (Fold change = 1 indicates same levels of gene expression as in controls. Fold-change > 1 indicates increased gene expression, while < 1 denotes decreased expression compared to controls). **a** In the substantia nigra, IL-1β was significantly upregulated in the PD group compared to controls (two-tailed unpaired t-test, t(17) = 2.263, *p* = 0.037; Control n = 5, PD n = 14). **b**, **c** No differences in cytokine expression were observed in the amygdala (two-tailed unpaired t-test, *p* > 0.05; Control = 5, PD = 8) and the hippocampus (two-tailed unpaired t-test, *p* > 0.05; Control n = 5, PD n = 13). **d** In the frontal cortex of PD cases, IL-1β was more highly expressed compared to controls (two-tailed unpaired t-test, t(15) = 2.278, *p* = 0.038; Control n = 6, PD n = 11). Fold change = 2^−∆∆CT^. *p < 0.05

Quantitative real-time PCR with primers for TNFα, IL-1β, IL6, and IL8 was performed in four brain regions (substantia nigra, amygdala, hippocampus, and frontal cortex). Due to the limited availability of frozen brain tissue, PDD and PD cases were collapsed into a single group for comparison with controls. In the substantia nigra and frontal cortex, there was a significant increase in the expression of the pro-inflammatory cytokine IL-1β in PD compared to controls (*p* < 0.05) (Fig. 6a and d). In the amygdala and hippocampus there were no between-group differences in cytokine expression (Fig. 6b, c).

### TLR4 expression is upregulated in the substantia nigra, amygdala and frontal cortex of PD brains

**Fig. 7:**
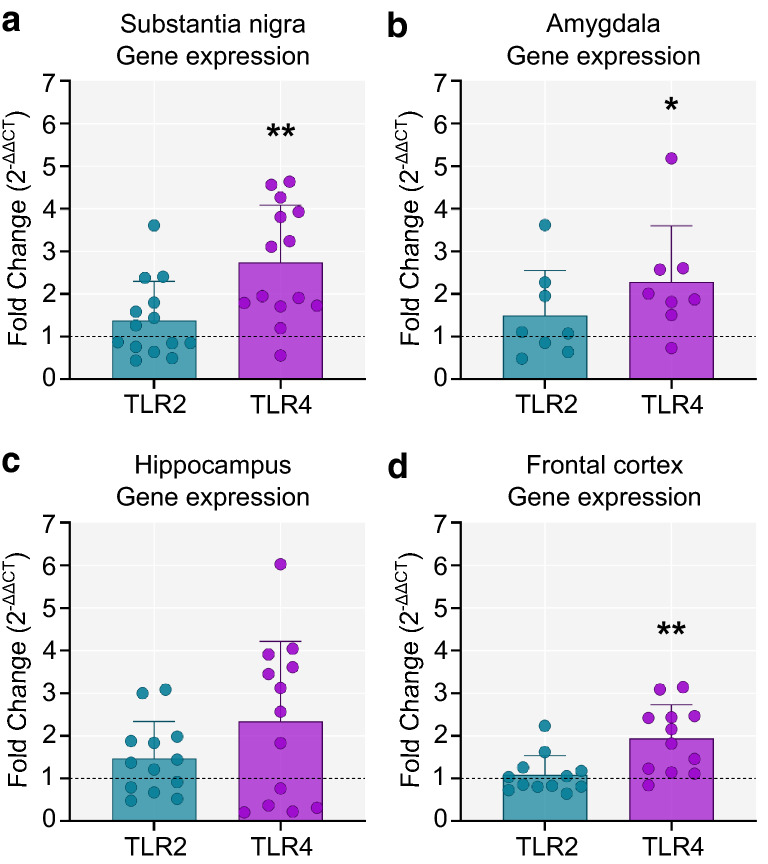
Expression of TLR2 and TLR4 in PD and control brains. Fold change in TLR2 and TLR4 gene expression in PD compared to controls. The dotted line marks the control group (Fold change = 1 indicates same levels of gene expression as in controls. Fold-change > 1 indicates increased gene expression, while < 1 denotes decreased expression compared to controls). **a** In the substantia nigra, TLR4 expression was significantly higher in the PD group compared to controls (two-tailed unpaired t-test, t(18) = 3.09 *p* = 0.006; Control n = 6, PD n = 14) **b** TLR4 expression was also elevated in the amygdala (two-tailed unpaired t-test, t(11) = 2.41, *p* = 0.035; Control n = 5, PD n = 8). **c** No differences in either TLR2 or TLR4 gene expression were observed in the hippocampus (two-tailed unpaired t-test *p* > 0.05; Control n = 7, PD n = 13). **d** There was a significant increase in TLR4 expression in the frontal cortex of PD cases compared to controls (two-tailed unpaired t-test, t(17) = 3.14, *p* = 0.006; Control n = 7, PD n = 12). Fold change = 2^−∆∆CT^. *p < 0.05, **p < 0.01

Quantitative real-time PCR with primers for TLR2 and TLR4 was performed in four brain regions (substantia nigra, amygdala, hippocampus, and frontal cortex). TLR2 expression was similar in PD and controls across in all four brain regions (Fig. 7). In contrast, TLR4 was significantly upregulated in the substantia nigra (*p* = 0.006), the amygdala (*p* = 0.035) and the frontal cortex (*p* = 0.006) of PD cases compared to controls (Fig. 7).

### Correlation between neuropathological and immune markers

Spearman’s rank-order correlation in PD cases between the pathological proteins in each brain region revealed a consistent association between α-synuclein and tau in all the examined brain regions, most strongly in the amygdala (Rho = 0.575, *p* = 0.003) and prefrontal cortex (Rho = 0.602, *p* = 0.003) (Table 2). There was also a correlation between α-synuclein and amyloid-β in the occipitotemporal and posterior parietal cortices. No significant correlation was observed between tau and amyloid-β in any brain region.

**Table 2:** Spearman’s rank-order correlation between pathological proteins in PD cases

Region		Tau		Amyloid-β	
		Rho	p value	Rho	p value
Amygdala (n = 24)	α-synuclein	0.575	0.003**	0.219	0.304
Hippocampus (n = 22)		0.431	0.040*	0.034	0.879
Entorhinal cortex (n = 23)		0.468	0.024*	0.257	0.248
Occipitotemporal cortex (n = 22)		0.535	0.010*	0.438	0.047*
Prefrontal cortex (n = 22)		0.602	0.003**	0.385	0.077
Posterior parietal cortex (n = 28)		0.354	0.070*	0.413	0.029*

*Rho* Spearman’s correlation coefficient

*p < 0.05; **p < 0.01

In PDND and PDD cases, Spearman’s rank-order correlation was performed between all three pathological proteins and the number of activated microglia in each brain region (Supplementary Table 1). In the amygdala, HLA-DR^+^ microglial count significantly correlated with α-synuclein (Rho = 0.448; *p* = 0.028). In the posterior parietal cortex, the number of activated microglia was significantly associated with tau pathology (Rho = 0.471; *p* = 0.013). No correlation between activated microglia and amyloid-β was observed in any region.

In the amygdala, Spearman’s rank-order correlation also revealed a significant correlation between CD4^+^ T lymphocytes with both α-synuclein (Rho = 0.443; *p* = 0.034) and tau pathology (Rho = 0.420; *p* = 0.046), as well as a correlation between CD8^+^ T lymphocytes and tau pathology (Rho = 0.569; *p* = 0.006) (Supplementary Table 2). In this brain region, the number of infiltrating CD4^+^, but not CD8^+^ T lymphocytes was correlated with the number of activated microglia (Spearman’s Rho = 0.525; *p* = 0.01).

### Association between neuropathology and cognitive decline

We sought to identify which pathological marker was the best correlate to longitudinal cognitive decline in life (measured by the MMSE). Only cases with clinical data collected up to and including 3 years prior to death were included in this analysis (n = 13). Four variables correlated significantly with MMSE change per year using Spearman’s Rank-Order Correlation (i.e., α-synuclein pathology in the hippocampus, entorhinal, occipitotemporal and prefrontal cortices). Univariate linear regression, correcting for age at death and disease duration, confirmed a significant association between MMSE change per year and α-synuclein pathology in the hippocampus (F_(3,9)_ = 14.73, *p* = 0.001), entorhinal (F_(3,9)_ = 33.98, *p* < 0.0001), occipitotemporal (F_(3,9)_ = 7.80, *p* = 0.007), and prefrontal cortex (F_(3,9)_ = 12.51, *p* = 0.002) (Table 3).

**Table 3:** Univariate linear regression of MMSE change per year with correction for age at death and disease duration

Variable	Standardized beta coefficient	Adjusted R^2^	*p* value
α-Synuclein in the hippocampus	− 0.914	0.774	0.001
α-Synuclein in the entorhinal cortex	− 0.972	0.892	< 0.0001
α-Synuclein in the occipitotemporal cortex	− 0.868	0.630	0.007
α-Synuclein in the prefrontal cortex	− 0.958	0.738	0.002

*MMSE* Mini-mental state examination

## Discussion

This study provides novel insights into the neuropathological substrates of cognitive decline in PD through investigating, for the first time, the nature and distribution of neuroinflammatory change in PD cases with or without dementia and correlating this with protein pathology. We confirm previous findings that α-synuclein pathology correlates with the rate of cognitive decline in PD, whilst the levels of Alzheimer’s disease-type pathology were found to be comparable across groups. Neuroinflammatory change in PDD cases was most pronounced in the amygdala, a limbic region heavily implicated in emotion and cognition [61]. Specifically, we observed increased microglial activation in the amygdala in PDD brains compared to controls and found evidence of CD4^+^ T cell infiltration into this region in all PD dementia cases. Furthermore, microglial activation, CD4^+^ T cell infiltration and α-synuclein pathology were correlated in this region, implicating an α-synuclein-driven neuroinflammatory response in the amygdala in PD dementia. We explored the expression of pro-inflammatory cytokines as well as TLR2 and TLR4 in several extra-nigrostriatal regions and observed elevated expression of TLR4 in the amygdala, frontal cortex, and substantia nigra, accompanied by elevated levels of the downstream inflammatory cytokine IL-1β. Taken together, our observations are consistent with the hypothesis that α-synuclein drives a neuroinflammatory response in PD through the activation of microglial TLR4 [46] and suggest a contributory role for peripheral T lymphocytes.

Our results show a significantly higher burden of α-synuclein pathology in PDD compared to PDND cases across multiple brain regions. This is consistent with previous findings of increased α-synuclein burden in PD dementia. Compta et al. using both a semi-quantitative Lewy body scoring system and quantification of Lewy body density per mm^2^ found a significantly elevated burden in PDD (n = 29) compared to PDND brains (n = 27), particularly in the frontal, temporal, cingulate and entorhinal cortex [9]. A second large autopsy study in 92 PDD and 48 PDND brains using traditional scoring protocols reported similar results in the same brain regions [38]. These findings were further validated in another large study with 55 PDD and 49 PDND cases [64]. Previous volumetric MRI studies have also implicated the amygdala in PD dementia showing significant atrophy of this region in demented PD patients compared to healthy controls but not in cognitively intact PD patients compared to controls, thus implicating the amygdala in cognitive decline in PD [40]. A limited number of studies have also addressed the role of abnormalities in the amygdala in relation to other non-motor symptoms of PD. On a functional level, a magnetic resonance imaging (MRI) study revealed that in the absence of structural alterations, there were abnormally high levels of activity in the amygdala of depressed PD patients compared to patients without depression and to controls. This heightened activity was found to be positively correlated with clinical scores of depression. Functional connectivity between the amygdala and fronto-parietal cortices was also found to be reduced, specifically in the patients suffering from depression [32]. Furthermore, an early clinicopathological study showed that PD patients suffering from hallucinations had nearly double Lewy body density in the basolateral amygdala compared to patients that did not experience them [28]. Amygdala abnormalities have also been linked to cognitive decline in Alzheimer’s disease. Similar to PD, atrophy of the amygdala was shown to be substantial in two large independent cohorts of mild Alzheimer’s disease. The magnitude of atrophy was strongly predictive of cognitive decline as shown by a robust correlation with MMSE scores [62]. The role of amygdala dysfunction in PD dementia has not been extensively studied and based on our present findings may warrant further investigation.

Notably, we did not observe any significant differences in tau pathology between PDND and PDD cases. In agreement, semi-quantitative scoring in a large postmortem study revealed similar levels or neurofibrillary tau tangles in the temporal, mid-frontal, and parietal cortex of PDND and PDD cases [38]. Tau Braak staging, however, has shown inconsistent results across studies; whilst Horvath et al. found the overall tau Braak stage to be significantly higher in demented compared to non-demented PD cases [30], Ruffmann and colleagues did not find differences between the groups, with 84% of all cases having only mild tau pathology (Braak stage 0–2) [64]. We did not observe significant differences in amyloid-β pathology between controls, PDND, and PDD cases. In contrast, previous studies have reported higher amyloid-β scores in the hippocampus, striatum, entorhinal and frontal cortex of demented compared to non-demented PD brains [64]. The total amyloid-β plaque score, total amyloid angiopathy in the cortex [9], and the amyloid-β Thal phases [30, 72] have also been reported to be significantly higher in PDD compared to PDND. This discrepancy of our results with previous studies may be due to the difference in brain regions under investigation and the smaller sample size used in our study. Indeed, we observed a trend for increased amyloid-β deposition in the PDD cases compared to both PDND and controls, however this was not significant after correcting for multiple comparisons.

We hypothesized that neuroinflammation might be an additional neuropathological substrate contributing to dementia in PD. PET neuroimaging studies using [^11^C]PK11195, a ligand for TSPO which is upregulated on activated microglia, have similarly suggested that microglial activation is increased in PDD cases. Edison and colleagues demonstrated increased tracer uptake in multiple brain regions in demented PD patients compared to controls, which was much more widespread than in non-demented PD patients versus controls [20]. We have previously shown that PD patients with a higher risk of progressing to dementia have increased activation of the innate immune system, including an increase in classical (inflammatory) monocytes, and increased monocyte expression of both TLR2 and TLR4 compared to patients at low risk of dementia [74]. Furthermore, we found that a pro-inflammatory cytokine profile in the serum in newly-diagnosed PD patients was associated with faster UPDRS-III progression and more impaired cognitive function over 3 years of follow-up [77]. However, the contribution of neuroinflammation to PDD has not previously been explored at postmortem. Our novel data show an increase in activated HLA-DR^+^ microglia in the amygdala of PDD cases. In a previous study Imamura et al. showed increased numbers of HLA-DR^+^ microglia in the hippocampus, transentorhinal, cingulate and temporal cortex in a relatively small number of PD (n = 12) and control (n = 4) autopsy cases, though in this study no distinction was made between demented and non-demented PD brains [36]. The lack of a PD versus control difference in activated microglia in these regions in our study may relate to the characteristics of the control population used. We opted to select typical elderly controls on the basis of having no neurological or cognitive symptoms during life and not on the basis of an absence of tau or amyloid-β pathology in the brain. In contrast, other authors typically select “supranormal” controls with no neurofibrillary tau tangles or amyloid-β plaques. Such controls are not representative of the normal neurologically intact aged population; indeed it has been repeatedly demonstrated that misfolded tau and amyloid-β accumulation occurs during ageing in the absence of neurodegenerative disease [3, 11, 50, 63, 65]. Our controls had a degree of amyloid-β and tau pathology and such misfolded protein deposition may trigger low level microglial activation. This could explain the contradictory findings in our study compared to previous work [36].

Surprisingly, we did not find a difference in HLA-DR^+^ microglia count in the substantia nigra of PD cases (either PDND or PDD) compared to controls. This is in contrast to the seminal study by McGeer et al. in 1988 who first reported an increase in HLA-DR^+^ microglia in this region of PD cases compared to controls [52]. Similar findings have been reported by a subsequent study showing an increase in both amoeboid CD68^+^ microglia as well as Iba1^+^ microglia in the postmortem PD nigra [15]. These inconsistencies with our findings may be partly explained by methodological differences in the identification of activated microglia as discussed below, as well as differences in the selection of control populations. [^11^C]PK11195 PET neuroimaging studies have also shown conflicting data. Ouchi et al. found increased binding in the midbrain of newly diagnosed PD patients [59], whilst Gerhard et al. did not find a difference in the substantia nigra of patients compared to controls [25]. Additional studies are needed to ascertain the extent of microglial activation in the substantia nigra and at which stage of the disease this is more prominent.

Although we did not observe an increase in number of activated microglia in the nigra, we did observe increased infiltration of peripheral T lymphocytes in this region in PDD and PDND cases, as well as elevated IL-1β levels, providing alternative evidence of immune activation in this region. Brochard et al. have previously shown an increased number of both CD4^+^ and CD8^+^ T lymphocytes in the substantia nigra of PD cases compared to controls, especially in the vicinity of dopaminergic neurons [6]. Additionally, recent work by Sommer et al. using CD3, a pan-T lymphocyte marker, revealed an increase in total CD3^+^ T lymphocytes (including parenchymal and perivascular cells) in the substantia nigra of PD cases compared to controls [68]. Our results corroborate these earlier findings and we also show that this increase is predominantly seen in PD dementia cases compared to controls. Furthermore, we observed a similar non-significant trend in the amygdala, particularly in the numbers of CD4^+^ but not of CD8^+^ T lymphocytes, and found that significant CD4^+^ T lymphocyte infiltration (more than 10 cells) into the amygdala was more common in PDND and PDD (50% and 33% of cases) compared to controls (10% of cases). Infiltration of T lymphocytes in the brain parenchyma has also been observed in other synucleinopathies. In particular, CD4^+^ but not CD8^+^ or B lymphocytes were found to be increased in the frontal cortex and hippocampus of cases with dementia with Lewy bodies (DLB) compared to controls at postmortem [35]. A second recent study in DLB cases showed increased T lymphocyte infiltration in both the grey and white matter of the middle temporal gyrus, in the absence of prominent microglial activation [2]. Similarly, in the substantia nigra of cases with multiple system atrophy compared to controls both CD4^+^ and CD8^+^ T lymphocytes were found to be increased [75]. The role of infiltrating T lymphocytes in PD is still unclear, however, ablation of CD4^+^ T cells in an MPTP mouse model of PD was found to be neuroprotective [6]. In another set of experiments using an AAV-α-synuclein rat model of PD it was observed that T cell-deficient (athymic nude) mice were protected from dopaminergic neuron loss in the substantia nigra [70]. Taken together, this data suggests that these adaptive immune cells may have a cytotoxic effect in PD and related synucleinopathies. Furthermore, recent evidence from human studies suggests that α-synuclein epitopes are recognised by autoreactive CD4^+^ T lymphocytes in PD [71], which may explain our observed a significant correlation between CD4^+^ T cells and α-synuclein pathology in the amygdala in our PD cases.

Both activated microglia and infiltrating lymphocytes may be exerting neurotoxic effects via the production of pro-inflammatory cytokines. In this study, we report for the first time an upregulation of the pro-inflammatory cytokine IL-1β in the frontal cortex of PD cases compared to controls. Gene expression of IL-1β was also increased in the PD substantia nigra, in line with previous evidence [55]. It should, however, be noted that caution is needed when interpreting these results, given that the control sample size available for gene expression analysis was small. Furthermore, bulk tissue was used in these experiments with normalisation against housekeeping genes. Therefore, the reported gene expression findings have not been adjusted for potential differences in the ratio of neurons to glial cells which may occur due to increased neuron loss in certain regions in the PD cases compared to controls. The expression of pro-inflammatory cytokines in postmortem PD has not been extensively investigated in the past. In fact, previously available data come primarily from early work by Mogi and colleagues who quantified the protein levels of several cytokines in the substantia nigra using enzyme-linked immunoassays. They reported higher levels of both IL-1β and IL6 [55], as well as elevated TNFα and IL2 in the substantia nigra of PD brains compared to controls [56, 57].

One likely pathway leading to upregulation and secretion of pro-inflammatory cytokines is that mediated by Toll-like receptor activation. In vitro experiments have shown that microglia can be directly activated by misfolded α-synuclein through both TLR2 [44] and TLR4 [22, 66]. Increased protein levels of TLR2 and TLR4 have previously been reported in the substantia nigra [15, 67], and the caudate/putamen of PD compared to control brains [16], and in addition, these receptors are upregulated in peripheral blood mononuclear cells of PD patients compared to controls [16, 74]. Our work has now shown elevation of TLR4 expression in multiple brain regions including the substantia nigra, amygdala and frontal cortex of PD cases compared to controls. Notably, in these same brain regions in PD, we also observed increased expression of IL-1β, a downstream product of the inflammasome pathway which is triggered by TLR4 activation. Interestingly, TLR2 and TLR4 have also been implicated in other proteinopathies, including Alzheimer’s [69] and Huntington’s disease [73] raising the possibility of a common pathogenic mechanism across several neurodegenerative diseases. The concomitant increase in the gene expression of TLR4 and IL-1β in the substantia nigra and the frontal cortex suggests an involvement of the NOD-like receptor protein 3 (NLRP3) inflammasome in these regions, with TLR4 activation resulting in increased expression of pro-IL-1β as well as NLRP3 activation; in turn, NLRP3 inflammasome activation could be responsible for the cleavage of pro-IL1β to the mature protein. This hypothesis is supported by recent data showing an upregulation of the protein levels of the NLRP3 adapter protein ASC (apoptosis-associated speck-like protein containing a caspase recruitment domain) as well as cleaved caspase-1 in postmortem nigral samples of PD cases compared to controls [26]. The same study also showed that inhibition of NLRP3 activation in a mouse model of PD (intrastriatal injection of α-synuclein pre-formed fibrils) could effectively mitigate motor dysfunction as well as dopaminergic neuron loss. Nevertheless, future studies are necessary to determine whether NLPR3 inflammasome activation is also occurring in other extranigral regions, particularly in the amygdala and the frontal cortex of PD cases versus controls.

In this study, we observed significant correlations between cortical α-synuclein Lewy pathology and the rate of cognitive decline during life. This finding corroborates previous evidence showing a robust correlation between cortical Lewy body burden and cognitive decline in PD using multivariate linear regression analyses [1, 64]. Other investigators have used the presence of dementia as the primary outcome in a logistic regression model, showing that neocortical Lewy body burden is strongly associated with dementia in PD [30, 38]. In keeping with our findings that tau and amyloid-β pathology did not differ between demented and non-demented PD cases, we did not find a correlation between either tau or amyloid-β in any of the examined brain regions and MMSE decline per year.

Although our data implicate neuroinflammation, particularly in the amygdala in PDD, we did not find a significant correlation between inflammatory changes in this region and cognitive decline during life. A previous study similarly found no correlation between HLA-DR^+^ microglia in the substantia nigra and clinical parameters in PD, whilst the use of a different marker CD68 (indicative of microglial phagocytic activity) revealed a strong association between CD68^+^ microglia and disease duration [12]. The method of characterizing activated microglia in postmortem brain may be critical to revealing clinicopathological correlations.

Indeed, a limitation of our study and a major challenge in postmortem brain studies overall is the definition of “activated microglia”. Here we have used enlarged and amoeboid morphology to quantify activated microglia selectively. However, this is a subjective method and microglial morphology is not restricted into either ramified or amoeboid shapes but represents a continuum including a whole range of morphological phenotypes [7]. Another caveat in our analysis is that the immunostaining of microglia was performed on thin brain sections  (10 µm). Previous studies assessing phenotypic differences to classify microglia have done so in sections 30–40 µm-thick [24, 48], with Kongsui and colleagues finding that the diameter of many microglia ranges between 40 and 50 µm [45] suggesting that even thicker sections would be needed for morphological studies. Furthermore, although microglia had been generally considered to be a functionally homogeneous population, comprehensive RNA sequencing studies have found evidence of different microglial subtypes with distinct function and have identified markers which can help distinguish between them. For instance, a recent single-cell RNA sequencing study in postmortem brain tissue from Alzheimer’s disease cases discovered a subtype of “disease-associated microglia” (DAM), with a unique transcriptional and functional profile, characterized by high phagocytic activity and upregulation of specific markers such as TREM2 [43]. Therefore, future work in postmortem brain could make use of additional markers such as P2RY12 and TMEM119 (homeostatic microglia) [4], and TREM2 (DAM) to better understand the role of microglia in PD. Future work could also utilise comprehensive genome-wide expression analyses which have become possible through the use of protocols to isolate microglia from human brain tissue [58] or by singe-cell RNA sequencing using bulk tissue, as has recently been done in Alzheimer’s disease postmortem brain [14].

Strengths of our study include our cohort of clinically well-characterised cases enabling the correlation between the pathological markers with the clinical course of the disease during life, as well as the use of controls who are representative of the typical aged population as discussed earlier. Additionally, in this study we employed a digital image analysis approach to quantitatively evaluate the severity of protein pathology in the postmortem brain. Traditional pathology is based on semiquantitative scoring upon visual inspection [53, 54] and is a useful method of assessing the distribution and overall protein pathology burden but it is inevitably subject to inter-rater variability and may also lack sensitivity particularly in identifying subtle differences in pathology severity. Digital quantification is a reliable alternative, useful for high throughput analysis, and can provide a more accurate quantitative measure of pathology severity [18, 23]. The methodology used in the present study was based on work described by Dunn et al. who showed a strong correlation between the automated analysis and the conventional scoring methodology [18].

In summary, this study demonstrates that dementia in PD is associated with increased neuroinflammation in the substantia nigra and amygdala at postmortem, involving microglial activation and the infiltration of T lymphocytes. We also report an upregulation of the pro-inflammatory cytokine IL-1β and upstream TLR4 in both the substantia nigra and extra-nigrostriatal regions in PD. We have confirmed that limbic and neocortical α-synuclein is the most robust predictor for dementia in PD and identified a correlation between α-synuclein and neuroinflammation in the amygdala. Taken together, this data suggests that a combination of α-synuclein pathology and inflammatory changes in the brain are critically involved in dementia in PD.

## Supplementary information

**Additional file 1**: **Fig. S1. Iba1**^**+**^ **activated microglia in the substantia nigra**. (**a**) Representative image of Iba1^+^ microglia in the substantia nigra of a control (left) and a Parkinson’s brain (right). The dark brown pigmented cells are neuromelanin-containing dopaminergic neurons. (**b**) Quantification of the total activated (enlarged amoeboid) microglia per mm^2^ (Kruskal–Wallis with Dunn’s multiple comparisons test, p = 0.269). Control n = 12, PDND n = 15, PDD n = 8. PDND: Parkinson’s disease no dementia, PDD: Parkinson’s disease dementia. Scale bar: 100 μm. *p < 0.05.

**Additional file 2**: **Fig. S2. CD3**^**+**^ **T lymphocytes in the substantia nigra**. (**a**) Representative image of parenchyma infiltrating CD3^+^ T lymphocytes in the substantia nigra of a control (left) and a Parkinson’s brain (right). The dark brown pigment is neuromelanin within dopaminergic neurons; the smaller CD3^+^ T cells are shown in the higher magnification inserts indicated by black squares. (**b**) Quantification of parenchymal CD3^+^ T lymphocytes per mm^2^ in the substantia nigra (Mann–Whitney U test, p = 0.038). Control n = 7, PD n = 16. Scale bar: 100 μm. Scale bar (insert): 20 μm. *p < 0.05.

**Additional file 3**: Spearman’s rank-order correlation between pathological proteins and activated microglia.

**Additional file 4**: Spearman’s rank-order correlation between pathological proteins and infiltrating T lymphocytes in the amygdala.

## Data Availability

Supporting data related to the findings of this study will be made available by the authors upon reasonable request by suitably qualified investigators.

## References

[1] Aarsland D, Perry R, Brown A, Larsen JP, Ballard C (2005). Neuropathology of dementia in Parkinson’s disease: a prospective, community-based study. Ann Neurol.

[2] Amin J, Holmes C, Dorey RB, Tommasino E, Casal YR, Williams DM, Dupuy C, Nicoll JAR, Boche D (2020). Neuroinflammation in dementia with Lewy bodies: a human post-mortem study. Transl Psychiatry.

[3] Bennett DA, Schneider JA, Arvanitakis Z, Kelly JF, Aggarwal NT, Shah RC, Wilson RS (2006). Neuropathology of older persons without cognitive impairment from two community-based studies. Neurology.

[4] Bennett ML, Bennett FC, Liddelow SA, Ajami B, Zamanian JL, Fernhoff NB, Mulinyawe SB, Bohlen CJ, Adil A, Tucker A (2016). New tools for studying microglia in the mouse and human CNS. Proc Natl Acad Sci.

[5] Braak H, Rüb U, Steur ENHJ, Tredici KD, de Vos RAI (2005). Cognitive status correlates with neuropathologic stage in Parkinson disease. Neurology.

[6] Brochard V, Combadière B, Prigent A, Laouar Y, Perrin A, Beray-Berthat V, Bonduelle O, Alvarez-Fischer D, Callebert J, Launay J-M, Duyckaerts C, Flavell RA, Hirsch EC, Hunot S (2008). Infiltration of CD4^+^ lymphocytes into the brain contributes to neurodegeneration in a mouse model of Parkinson disease. J Clin Invest.

[7] Cherry JD, Olschowka JA, O’Banion MK (2014). Neuroinflammation and M2 microglia: the good, the bad, and the inflamed. J Neuroinflammation.

[8] Colosimo C, Hughes AJ, Kilford L, Lees AJ (2003). Lewy body cortical involvement may not always predict dementia in Parkinson’s disease. J Neurol Neurosurg Psychiatry.

[9] Compta Y, Parkkinen L, O’Sullivan SS, Vandrovcova J, Holton JL, Collins C, Lashley T, Kallis C, Williams DR, de Silva R, Lees AJ, Revesz T (2011). Lewy- and Alzheimer-type pathologies in Parkinson’s disease dementia: which is more important?. Brain.

[10] Coughlin D, Xie SX, Liang M, Williams A, Peterson C, Weintraub D, McMillan CT, Wolk DA, Akhtar RS, Hurtig HI, Branch Coslett H, Hamilton RH, Siderowf AD, Duda JE, Rascovsky K, Lee EB, Lee VM-Y, Grossman M, Trojanowski JQ, Irwin DJ (2018). Cognitive and pathological influences of tau pathology in lewy body disorders. Ann Neurol.

[11] Crary JF, Trojanowski JQ, Schneider JA, Abisambra JF, Abner EL, Alafuzoff I, Arnold SE, Attems J, Beach TG, Bigio EH, Cairns NJ, Dickson DW, Gearing M, Grinberg LT, Hof PR, Hyman BT, Jellinger K, Jicha GA, Kovacs GG, Knopman DS, Kofler J, Kukull WA, Mackenzie IR, Masliah E, McKee A, Montine TJ, Murray ME, Neltner JH, Santa-Maria I, Seeley WW, Serrano-Pozo A, Shelanski ML, Stein T, Takao M, Thal DR, Toledo JB, Troncoso JC, Vonsattel JP, White CL, Wisniewski T, Woltjer RL, Yamada M, Nelson PT (2014). Primary age-related tauopathy (PART): a common pathology associated with human aging. Acta Neuropathol (Berl).

[12] Croisier E, Moran LB, Dexter DT, Pearce RK, Graeber MB (2005). Microglial inflammation in the parkinsonian substantia nigra: relationship to alpha-synuclein deposition. J Neuroinflammation.

[13] Daniele SG, Béraud D, Davenport C, Cheng K, Yin H, Maguire-Zeiss KA (2015). Activation of MyD88-dependent TLR1/2 signaling by misfolded α-synuclein, a protein linked to neurodegenerative disorders. Sci Signal.

[14] Del-Aguila JL, Li Z, Dube U, Mihindukulasuriya KA, Budde JP, Fernandez MV, Ibanez L, Bradley J, Wang F, Bergmann K, Davenport R, Morris JC, Holtzman DM, Perrin RJ, Benitez BA, Dougherty J, Cruchaga C, Harari O (2019). A single-nuclei RNA sequencing study of Mendelian and sporadic AD in the human brain. Alzheimers Res Ther.

[15] Doorn KJ, Moors T, Drukarch B, van de Berg WD, Lucassen PJ, van Dam A-M (2014). Microglial phenotypes and toll-like receptor 2 in the substantia nigra and hippocampus of incidental Lewy body disease cases and Parkinson’s disease patients. Acta Neuropathol Commun.

[16] Drouin-Ouellet J, St-Amour I, Saint-Pierre M, Lamontagne-Proulx J, Kriz J, Barker RA, Cicchetti F (2015). Toll-like receptor expression in the blood and brain of patients and a mouse model of Parkinson’s disease. Int J Neuropsychopharmacol.

[17] Dubois B, Burn D, Goetz C, Aarsland D, Brown RG, Broe GA, Dickson D, Duyckaerts C, Cummings J, Gauthier S, Korczyn A, Lees A, Levy R, Litvan I, Mizuno Y, McKeith IG, Olanow CW, Poewe W, Sampaio C, Tolosa E, Emre M (2007). Diagnostic procedures for Parkinson’s disease dementia: recommendations from the movement disorder society task force. Mov Disord.

[18] Dunn WD, Gearing M, Park Y, Zhang L, Hanfelt J, Glass JD, Gutman DA (2016). Applicability of digital analysis and imaging technology in neuropathology assessment. Neuropathol Off J Jpn Soc Neuropathol.

[19] Dzamko N, Gysbers A, Perera G, Bahar A, Shankar A, Gao J, Fu Y, Halliday GM (2017). Toll-like receptor 2 is increased in neurons in Parkinson’s disease brain and may contribute to alpha-synuclein pathology. Acta Neuropathol (Berl).

[20] Edison P, Ahmed I, Fan Z, Hinz R, Gelosa G, Chaudhuri KR, Walker Z, Turkheimer FE, Brooks DJ (2013). Microglia, amyloid, and glucose metabolism in Parkinson’s disease with and without dementia. Neuropsychopharmacology.

[21] Emre M, Aarsland D, Brown R, Burn DJ, Duyckaerts C, Mizuno Y, Broe GA, Cummings J, Dickson DW, Gauthier S, Goldman J, Goetz C, Korczyn A, Lees A, Levy R, Litvan I, McKeith I, Olanow W, Poewe W, Quinn N, Sampaio C, Tolosa E, Dubois B (2007). Clinical diagnostic criteria for dementia associated with Parkinson’s disease. Mov Disord.

[22] Fellner L, Irschick R, Schanda K, Reindl M, Klimaschewski L, Poewe W, Wenning GK, Stefanova N (2013). Toll-like receptor 4 is required for α-synuclein dependent activation of microglia and astroglia. Glia.

[23] Ferman TJ, Aoki N, Crook JE, Murray ME, Graff-Radford NR, van Gerpen JA, Uitti RJ, Wszolek ZK, Graff-Radford J, Pedraza O, Kantarci K, Boeve BF, Dickson DW (2018). The limbic and neocortical contribution of α-synuclein, tau, and amyloid β to disease duration in dementia with Lewy bodies. Alzheimers Dement.

[24] del Fernández-Arjona MM, Grondona JM, Granados-Durán P, Fernández-Llebrez P, López-Ávalos MD (2017). Microglia morphological categorization in a rat model of neuroinflammation by hierarchical cluster and principal components analysis. Front Cell Neurosci.

[25] Gerhard A, Pavese N, Hotton G, Turkheimer F, Es M, Hammers A, Eggert K, Oertel W, Banati RB, Brooks DJ (2006). In vivo imaging of microglial activation with [11C](R)-PK11195 PET in idiopathic Parkinson’s disease. Neurobiol Dis.

[26] Gordon R, Albornoz EA, Christie DC, Langley MR, Kumar V, Mantovani S, Robertson AAB, Butler MS, Rowe DB, O’Neill LA, Kanthasamy AG, Schroder K, Cooper MA, Woodruff TM (2018). Inflammasome inhibition prevents α-synuclein pathology and dopaminergic neurodegeneration in mice. Sci Transl Med.

[27] Halliday G, Hely M, Reid W, Morris J (2008). The progression of pathology in longitudinally followed patients with Parkinson’s disease. Acta Neuropathol (Berl).

[28] Harding AJ, Stimson E, Henderson JM, Halliday GM (2002). Clinical correlates of selective pathology in the amygdala of patients with Parkinson’s disease. Brain.

[29] Hely MA, Reid WGJ, Adena MA, Halliday GM, Morris JGL (2008). The Sydney multicenter study of Parkinson’s disease: the inevitability of dementia at 20 years. Mov Disord.

[30] Horvath J, Herrmann FR, Burkhard PR, Bouras C, Kövari E (2013). Neuropathology of dementia in a large cohort of patients with Parkinson’s disease. Parkinsonism Relat Disord.

[31] Howlett DR, Whitfield D, Johnson M, Attems J, O’Brien JT, Aarsland D, Lai MKP, Lee JH, Chen C, Ballard C, Hortobágyi T, Francis PT (2015). Regional Multiple Pathology Scores Are Associated with Cognitive Decline in Lewy Body Dementias. Brain Pathol.

[32] Huang P, Xuan M, Gu Q, Yu X, Xu X, Luo W, Zhang M (2015). Abnormal amygdala function in Parkinson’s disease patients and its relationship to depression. J Affect Disord.

[33] Hunot S, Dugas N, Faucheux B, Hartmann A, Tardieu M, Debré P, Agid Y, Dugas B, Hirsch EC (1999). FcεRII/CD23 is expressed in Parkinson’s disease and induces, in vitro, production of nitric oxide and tumor necrosis factor-α in glial cells. J Neurosci.

[34] Hurtig HI, Trojanowski JQ, Galvin J, Ewbank D, Schmidt ML, Lee VM-Y, Clark CM, Glosser G, Stern MB, Gollomp SM, Arnold SE (2000). Alpha-synuclein cortical Lewy bodies correlate with dementia in Parkinson’s disease. Neurology.

[35] Iba M, Kim C, Sallin M, Kwon S, Verma A, Overk C, Rissman RA, Sen R, Sen JM, Masliah E (2020). Neuroinflammation is associated with infiltration of T cells in Lewy body disease and α-synuclein transgenic models. J Neuroinflammation.

[36] Imamura K, Hishikawa N, Sawada M, Nagatsu T, Yoshida M, Hashizume Y (2003). Distribution of major histocompatibility complex class II-positive microglia and cytokine profile of Parkinson’s disease brains. Acta Neuropathol (Berl).

[37] Irwin DJ, Grossman M, Weintraub D, Hurtig HI, Duda JE, Xie SX, Lee EB, Van Deerlin VM, Lopez OL, Kofler JK, Nelson PT, Jicha GA, Woltjer R, Quinn JF, Kaye J, Leverenz JB, Tsuang D, Longfellow K, Yearout D, Kukull W, Keene CD, Montine TJ, Zabetian CP, Trojanowski JQ (2017). Neuropathological and genetic correlates of survival and dementia onset in synucleinopathies: a retrospective analysis. Lancet Neurol.

[38] Irwin DJ, White MT, Toledo JB, Xie SX, Robinson JL, Van Deerlin V, Lee VM-Y, Leverenz JB, Montine TJ, Duda JE, Hurtig HI, Trojanowski JQ (2012). Neuropathologic substrates of Parkinson disease dementia. Ann Neurol.

[39] Jellinger KA, Seppi K, Wenning GK, Poewe W (2002). Impact of coexistent Alzheimer pathology on the natural history of Parkinson’s disease. J Neural Transm.

[40] Junqué C, Ramírez-Ruiz B, Tolosa E, Summerfield C, Martí M-J, Pastor P, Gómez-Ansón B, Mercader JM (2005). Amygdalar and hippocampal MRI volumetric reductions in Parkinson’s disease with dementia. Mov Disord.

[41] Kalaitzakis ME, Graeber MB, Gentleman SM, Pearce RKB (2008). Striatal β-Amyloid Deposition in Parkinson Disease With Dementia. J Neuropathol Exp Neurol.

[42] Kawai T, Akira S (2011). Toll-like receptors and their crosstalk with other innate receptors in infection and immunity. Immunity.

[43] Keren-Shaul H, Spinrad A, Weiner A, Matcovitch-Natan O, Dvir-Szternfeld R, Ulland TK, David E, Baruch K, Lara-Astaiso D, Toth B, Itzkovitz S, Colonna M, Schwartz M, Amit I (2017). A unique microglia type associated with restricting development of Alzheimer’s disease. Cell.

[44] Kim C, Ho D-H, Suk J-E, You S, Michael S, Kang J, Joong Lee S, Masliah E, Hwang D, Lee H-J, Lee S-J (2013). Neuron-released oligomeric α-synuclein is an endogenous agonist of TLR2 for paracrine activation of microglia. Nat Commun.

[45] Kongsui R, Beynon SB, Johnson SJ, Walker FR (2014). Quantitative assessment of microglial morphology and density reveals remarkable consistency in the distribution and morphology of cells within the healthy prefrontal cortex of the rat. J Neuroinflammation.

[46] Kouli A, Horne CB, Williams-Gray CH (2019). Toll-like receptors and their therapeutic potential in Parkinson’s disease and α-synucleinopathies. Brain Behav Immun.

[47] Kövari E, Gold G, Herrmann FR, Canuto A, Hof PR, Bouras C, Giannakopoulos P (2003). Lewy body densities in the entorhinal and anterior cingulate cortex predict cognitive deficits in Parkinson’s disease. Acta Neuropathol (Berl).

[48] Kozlowski C, Weimer RM (2012). An automated method to quantify microglia morphology and application to monitor activation state longitudinally in vivo. PLoS ONE.

[49] Livak KJ, Schmittgen TD (2001). Analysis of relative gene expression data using real-time quantitative PCR and the 2 − ΔΔCT method. Methods.

[50] Lowe VJ, Wiste HJ, Senjem ML, Weigand SD, Therneau TM, Boeve BF, Josephs KA, Fang P, Pandey MK, Murray ME, Kantarci K, Jones DT, Vemuri P, Graff-Radford J, Schwarz CG, Machulda MM, Mielke MM, Roberts RO, Knopman DS, Petersen RC, Jack CR (2018). Widespread brain tau and its association with ageing, Braak stage and Alzheimer’s dementia. Brain.

[51] Mattila PM, Rinne JO, Helenius H, Dickson DW, Röyttä M (2000). Alpha-synuclein-immunoreactive cortical Lewy bodies are associated with cognitive impairment in Parkinson’s disease. Acta Neuropathol (Berl).

[52] McGeer PL, Itagaki S, Boyes BE, McGeer EG (1988). Reactive microglia are positive for HLA-DR in the substantia nigra of Parkinson’s and Alzheimer’s disease brains. Neurology.

[53] McKeith IG, Dickson DW, Lowe J, Emre M, O’Brien JT, Feldman H, Cummings J, Duda JE, Lippa C, Perry EK, Aarsland D, Arai H, Ballard CG, Boeve B, Burn DJ, Costa D, Del Ser T, Dubois B, Galasko D, Gauthier S, Goetz CG, Gomez-Tortosa E, Halliday G, Hansen LA, Hardy J, Iwatsubo T, Kalaria RN, Kaufer D, Kenny RA, Korczyn A, Kosaka K, Lee VMY, Lees A, Litvan I, Londos E, Lopez OL, Minoshima S, Mizuno Y, Molina JA, Mukaetova-Ladinska EB, Pasquier F, Perry RH, Schulz JB, Trojanowski JQ, Yamada M, for the Consortium on DLB (2005). Diagnosis and management of dementia with Lewy bodies: third report of the DLB consortium. Neurology.

[54] Mirra SS, Heyman A, McKeel D, Sumi SM, Crain BJ, Brownlee LM, Vogel FS, Hughes JP, van Belle G, Berg L (1991). The Consortium to Establish a Registry for Alzheimer’s disease (CERAD). Part II. Standardization of the neuropathologic assessment of Alzheimer’s disease. Neurology.

[55] Mogi M, Harada M, Kondo T, Riederer P, Inagaki H, Minami M, Nagatsu T (1994). Interleukin-1β, interleukin-6, epidermal growth factor and transforming growth factor-α are elevated in the brain from parkinsonian patients. Neurosci Lett.

[56] Mogi M, Harada M, Kondo T, Riederer P, Nagatsu T (1996). Interleukin-2 but not basic fibroblast growth factor is elevated in parkinsonian brain. J Neural Transm.

[57] Mogi M, Harada M, Riederer P, Narabayashi H, Fujita K, Nagatsu T (1994). Tumor necrosis factor-α (TNF-α) increases both in the brain and in the cerebrospinal fluid from parkinsonian patients. Neurosci Lett.

[58] Olah M, Raj D, Brouwer N, Haas AHD, Eggen BJL, Dunnen WFAD, Biber KPH, Boddeke HWGM (2012). An optimized protocol for the acute isolation of human microglia from autopsy brain samples. Glia.

[59] Ouchi Y, Yoshikawa E, Sekine Y, Futatsubashi M, Kanno T, Ogusu T, Torizuka T (2005). Microglial activation and dopamine terminal loss in early Parkinson’s disease. Ann Neurol.

[60] Parkkinen L, Kauppinen T, Pirttilä T, Autere JM, Alafuzoff I (2005). α-Synuclein pathology does not predict extrapyramidal symptoms or dementia. Ann Neurol.

[61] Phelps EA (2005). Emotion and cognition: insights from studies of the human amygdala. Annu Rev Psychol.

[62] Poulin SP, Dautoff R, Morris JC, Barrett LF, Dickerson BC (2011). Amygdala atrophy is prominent in early Alzheimer’s disease and relates to symptom severity. Psychiatry Res.

[63] Rowe CC, Ng S, Ackermann U, Gong SJ, Pike K, Savage G, Cowie TF, Dickinson KL, Maruff P, Darby D, Smith C, Woodward M, Merory J, Tochon-Danguy H (2007). Imaging β-amyloid burden in aging and dementia. Neurology.

[64] Ruffmann C, Calboli FCF, Bravi I, Gveric D, Curry LK, de Smith A, Pavlou S, Buxton JL, Blakemore AIF, Takousis P, Molloy S, Piccini P, Dexter DT, Roncaroli F, Gentleman SM, Middleton LT (2015). Cortical Lewy bodies and Aβ burden are associated with prevalence and timing of dementia in Lewy body diseases. Neuropathol Appl Neurobiol.

[65] Schöll M, Lockhart SN, Schonhaut DR, O’Neil JP, Janabi M, Ossenkoppele R, Baker SL, Vogel JW, Faria J, Schwimmer HD, Rabinovici GD, Jagust WJ (2016). PET imaging of tau deposition in the aging human brain. Neuron.

[66] Shao Q-H, Yan W-F, Zhang Z, Ma K-L, Peng S-Y, Cao Y-L, Yuan Y-H, Chen N-H (2018). Nurr1: a vital participant in the TLR4-NF-κB signal pathway stimulated by α-synuclein in BV-2 cells. Neuropharmacology.

[67] Shin W-H, Jeon M-T, Leem E, Won S-Y, Jeong KH, Park S-J, McLean C, Lee SJ, Jin BK, Jung UJ, Kim SR (2015). Induction of microglial toll-like receptor 4 by prothrombin kringle-2: a potential pathogenic mechanism in Parkinson’s disease. Sci Rep.

[68] Sommer A, Maxreiter F, Krach F, Fadler T, Grosch J, Maroni M, Graef D, Eberhardt E, Riemenschneider MJ, Yeo GW, Kohl Z, Xiang W, Gage FH, Winkler J, Prots I, Winner B (2018). Th17 lymphocytes induce neuronal cell death in a human iPSC-based model of Parkinson’s disease. Cell Stem Cell.

[69] Su F, Bai F, Zhou H, Zhang Z (2016). Microglial toll-like receptors and Alzheimer’s disease. Brain Behav Immun.

[70] Subbarayan MS, Hudson C, Moss LD, Nash KR, Bickford PC (2020). T cell infiltration and upregulation of MHCII in microglia leads to accelerated neuronal loss in an α-synuclein rat model of Parkinson’s disease. J Neuroinflammation.

[71] Sulzer D, Alcalay RN, Garretti F, Cote L, Kanter E, Agin-Liebes J, Liong C, McMurtrey C, Hildebrand WH, Mao X, Dawson VL, Dawson TM, Oseroff C, Pham J, Sidney J, Dillon MB, Carpenter C, Weiskopf D, Phillips E, Mallal S, Peters B, Frazier A, Arlehamn CSL, Sette A (2017). T cells from patients with Parkinson’s disease recognize α-synuclein peptides. Nature.

[72] Thal DR, Rüb U, Schultz C, Sassin I, Ghebremedhin E, Del Tredici K, Braak E, Braak H (2000). Sequence of Abeta-protein deposition in the human medial temporal lobe. J Neuropathol Exp Neurol.

[73] Vuono R, Kouli A, Legault EM, Chagnon L, Allinson KS, La Spada A, REGISTRY Investigators of the European Huntington’s Disease Network, Biunno I, Barker RA, Drouin-Ouellet J (2019) Association between toll-like receptor 4 (TLR4) and triggering receptor expressed on myeloid cells 2 (TREM2) genetic variants and clinical progression of Huntington’s disease. Mov Disord Off J Mov Disord Soc. doi: 10.1002/mds.27911 10.1002/mds.27911 PMC7154663 31724242

[74] Wijeyekoon RS, Kronenberg-Versteeg D, Scott KM, Hayat S, Kuan W-L, Evans JR, Breen DP, Cummins G, Jones JL, Clatworthy MR, Andres Floto R, Barker RA, Williams-Gray CH (2020). Peripheral innate immune and bacterial signals relate to clinical heterogeneity in Parkinson’s disease. Brain Behav Immun.

[75] Williams GP, Marmion DJ, Schonhoff AM, Jurkuvenaite A, Won W-J, Standaert DG, Kordower JH, Harms AS (2020). T cell infiltration in both human multiple system atrophy and a novel mouse model of the disease. Acta Neuropathol (Berl).

[76] Williams-Gray CH, Mason SL, Evans JR, Foltynie T, Brayne C, Robbins TW, Barker RA (2013). The CamPaIGN study of Parkinson’s disease: 10-year outlook in an incident population-based cohort. J Neurol Neurosurg Psychiatry.

[77] Williams-Gray CH, Wijeyekoon R, Yarnall AJ, Lawson RA, Breen DP, Evans JR, Cummins GA, Duncan GW, Khoo TK, Burn DJ, Barker RA (2016). Serum immune markers and disease progression in an incident Parkinson’s disease cohort (ICICLE-PD). Mov Disord.

[78] Yuan JS, Reed A, Chen F, Stewart CN (2006). Statistical analysis of real-time PCR data. BMC Bioinformatics.

