# Supplementary figures and images for: Neuroinflammation and protein pathology in Parkinson’s disease dementia
Source: Acta Neuropathol Commun. 2020 Dec 3;8:211. doi: 10.1186/s40478-020-01083-5 (PMC7713145; doi:10.1186/s40478-020-01083-5)

**a**

# Substantia nigra

**Control****Parkinson's disease****Microglia (Iba1)**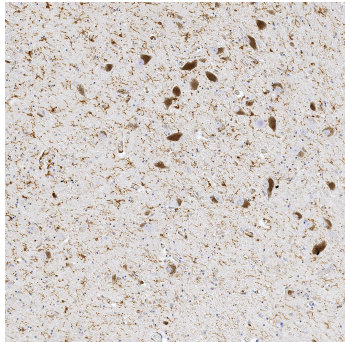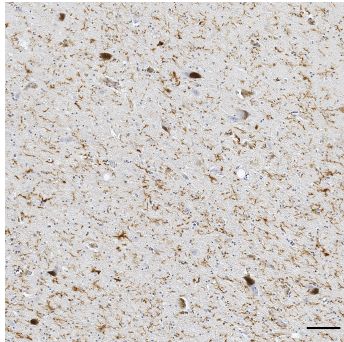**b****Microglial activation**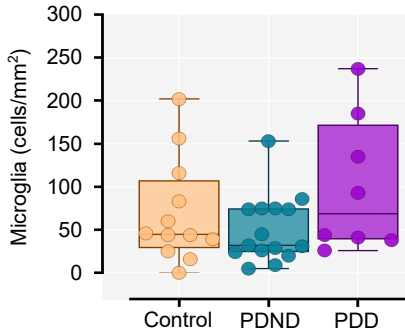

Supplement: Supplementary file 1 — Additional file 1: Fig. S1. Iba1+ activated microglia in the substantia nigra. (a) Representative image of Iba1+ microglia in the substantia nigra of a control (left) and a Parkinson’s brain (right). The dark brown pigmented cells are neuromelanin-containing dopaminergic neurons. (b) Quantification of the total activated (enlarged amoeboid) microglia per mm2 (Kruskal–Wallis with Dunn’s multiple comparisons test, p = 0.269). Control n = 12, PDND n = 15, PDD n = 8. PDND: Parkinson’s disease no dementia, PDD: Parkinson’s disease dementia. Scale bar: 100 μm. *p < 0.05. [file 40478_2020_1083_MOESM1_ESM.pdf]
