# Supplementary material for: Neuroinflammation and protein pathology in Parkinson’s disease dementia
Source: Acta Neuropathol Commun. 2020 Dec 3;8:211. doi: 10.1186/s40478-020-01083-5 (PMC7713145; doi:10.1186/s40478-020-01083-5)
Supplement: Supplementary file 2 — Additional file 2: Fig. S2. CD3+ T lymphocytes in the substantia nigra. (a) Representative image of parenchyma infiltrating CD3+ T lymphocytes in the substantia nigra of a control (left) and a Parkinson’s brain (right). The dark brown pigment is neuromelanin within dopaminergic neurons; the smaller CD3+ T cells are shown in the higher magnification inserts indicated by black squares. (b) Quantification of parenchymal CD3+ T lymphocytes per mm2 in the substantia nigra (Mann–Whitney U test, p = 0.038). Control n = 7, PD n = 16. Scale bar: 100 μm. Scale bar (insert): 20 μm. *p < 0.05. [file 40478_2020_1083_MOESM2_ESM.pdf]

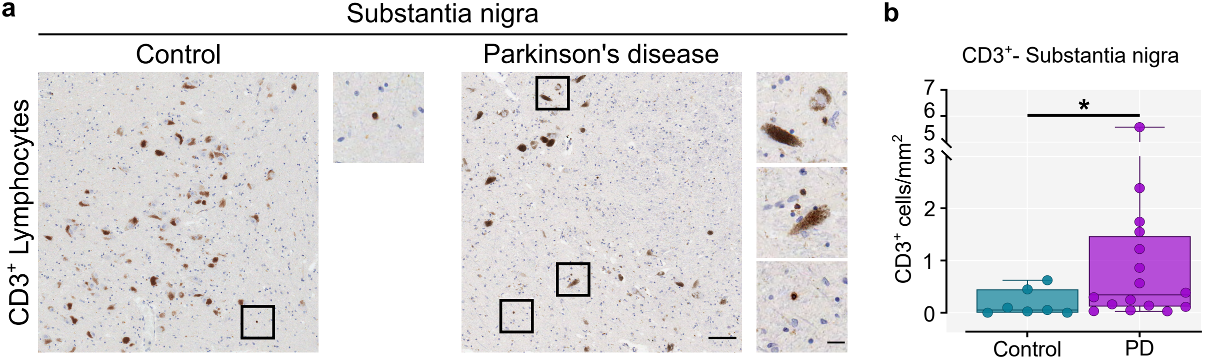

a
Substantia nigra
Control
Parkinson's disease
CD3+ Lymphocytes
b
CD3+- Substantia nigra
*
CD3+ cells/mm2
7
6
5
3
2
1
0
Control
PD
