# Supplementary material for: Neuroinflammation and protein pathology in Parkinson’s disease dementia
Source: Acta Neuropathol Commun. 2020 Dec 3;8:211. doi: 10.1186/s40478-020-01083-5 (PMC7713145; doi:10.1186/s40478-020-01083-5)
Supplement: Supplementary file 3 — Additional file 3: Spearman’s rank-order correlation between pathological proteins and activated microglia. [file 40478_2020_1083_MOESM3_ESM.pdf]

Supplementary Table 1. Spearman's rank-order correlation between pathological proteins and activated microglia.

Region		α-Synuclein		Tau		Amyloid-β	
		Rho	*p*	Rho	*p*	Rho	*p*
Amygdala (n=24)	Microglia	0.448	0.028*	0.165	0.442	0.282	0.182
Hippocampus (n=22)		0.410	0.052	-0.020	0.929	-0.027	0.907
Entorhinal cortex (n=23)		0.360	0.091	0.193	0.376	-0.287	0.195
Occipitotemporal cortex (n=22)		0.362	0.098	0.210	0.348	0.305	0.178
Prefrontal cortex (n=22)		-0.043	0.849	0.240	0.281	-0.173	0.441
Posterior parietal cortex (n=28)		0.085	0.668	0.471	0.013*	0.340	0.077

Rho: Spearman's correlation coefficient. *$p<0.05$.
