# Supplementary material for: Neuroinflammation and protein pathology in Parkinson’s disease dementia
Source: Acta Neuropathol Commun. 2020 Dec 3;8:211. doi: 10.1186/s40478-020-01083-5 (PMC7713145; doi:10.1186/s40478-020-01083-5)
Supplement: Supplementary file 4 — Additional file 4: Spearman’s rank-order correlation between pathological proteins and infiltrating T lymphocytes in the amygdala. [file 40478_2020_1083_MOESM4_ESM.pdf]

Supplementary Table 2. Spearman's rank-order correlation between pathological proteins and infiltrating T lymphocytes in the amygdala.

Pathological protein	$CD4^+$ lymphocytes		$CD8^+$ lymphocytes	
	Rho	*p*	Rho	*p*
α-Synuclein	0.443	0.034*	0.352	0.108
Tau	0.420	0.046*	0.569	0.006**
Amyloid-β	0.291	0.179	0.160	0.477

Rho: Spearman's correlation coefficient. n=23, *p<0.05, **p<0.01.
